# Isolated Microspherophakia Presenting with Angle-Closure Glaucoma

**DOI:** 10.4274/tjo.47135

**Published:** 2016-10-17

**Authors:** Tülay Şimşek, Emrullah Beyazyıldız, Enver Şimşek, Faruk Öztürk

**Affiliations:** 1 Osmangazi University Faculty of Medicine, Department of Ophthalmology, Eskişehir, Turkey; 2 Gazi Mustafa Kemal Hospital, Ophthalmology Clinic, Ankara, Turkey; 3 Osmangazi University Faculty of Medicine, Department of Pediatric Endocrinology, Eskişehir, Turkey; 4 Yıldırım Bayazıt University Faculty of Medicine, Department of Ophthalmology, Ankara, Turkey

**Keywords:** Microspherophakia, glaucoma, familial microspherophakia, lenticular myopia

## Abstract

We report a case of 13-year-old girl presenting to our clinic with blurred vision in both eyes. Ophthalmic examination revealed high myopia and angle-closure glaucoma due to pupillary block caused by small, spherical crystalline lenses. Treatment approaches to glaucoma in patients with microspherophakia are discussed in this case report.

## INTRODUCTION

Microspherophakia is a rare entity in which there is a small, spherical crystalline lens with increased antero-posterior thickness.^1^ The characteristic feature of microspherophakia is visibility of the lens equator on full mydriasis. The pathogenesis of this condition is thought to be related to defective development of the lens zonules.^2^ Spherical lens may lead to pupillary block and secondary angle-closure glaucoma. Glaucoma is the most common sight-threatening complication of this condition. Lenticular myopia and lens dislocation are other common findings of microspherophakia.^3^ Treatment of these patients is difficult and there is no consensus about the treatment approach, especially in patients presenting with secondary angle-closure glaucoma. We report a case with microspherophakia, whose brother also had microspherophakia, presenting as bilateral angle-closure glaucoma.

## CASE REPORT

**Figure 1:**
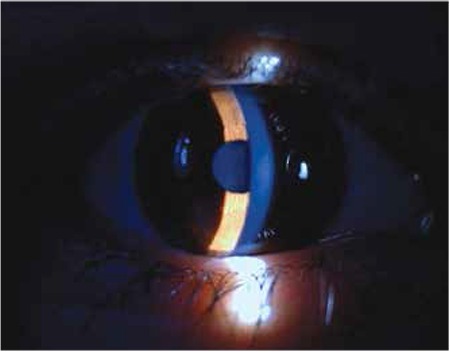
Slit-lamp biomicroscopic view of cornea and lens of the patient. Anterior chambers were shallow; lenses were thicker and steeper than normal and appeared to bulge forward into the pupil

**Figure 2:**
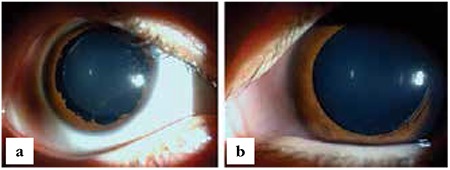
Slit-lamp biomicroscopic view of cornea and lens of the patient’s right (a) and left (b) eye after pupil dilation; lens edges and zonules were clearly visible

**Figure 3:**
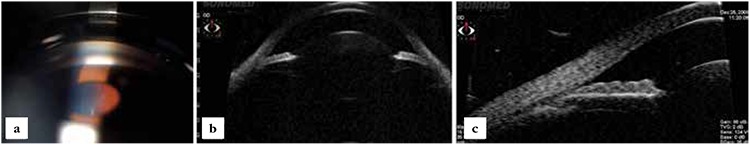
Gonioscopy (a) and ultrasound biomicroscopy showed an anteriorly displaced small and spheric crystalline lens (b) and almost 360 degree closed angles (c)

A 13-year-old girl presented to the ophthalmology clinic for refractive eye examination. Her intraocular pressure (IOP) was 38 mmHg in the right eye (OD) and 36 mmHg in the left eye (OS). She had no pain, lacrimation or blepharospams in her eyes. Her visual acuity was 20/20 with -12.0 DS/-3.00 DC x140 degrees in OD and 20/20 with -13.0 DS /-2.75 DC x160 degrees in OS. Central corneal thickness was 560 µm OD and 555 µm OS. Slit-lamp biomicroscopy revealed a shallow anterior chamber in both eyes. Lenses were thicker and steeper than normal and appeared to bulge forward into the pupil (Figure 1). The lens edges and long, weak zonules were clearly visible on slit-lamp examination after pupillary dilation (Figure 2a, 2b). Lens thickness after pupillary dilation was 4.93 mm OD and 4.96 mm OS. Gonioscopic examination revealed completely closed angles and no anterior synechia was observed with indentation. Ultrasound biomicroscopy (UBM) showed anteriorly displaced small and spheric crystalline lenses and almost 360 degree closed angles (Figure 3). Anterior chamber depth was 2.00 mm OD and 2.02 mm OS. Axial length was 20.23 mm OD and 20.28 mm OS, suggesting lenticular myopia. A thorough family history could not be obtained; however, she had a positive family history of high myopia and poor vision on her mother’s side.

Her brother also has myopia and microspherophakia. Her brother’s IOP was normal but appositional angle closure was observed in both eyes. No systemic anomalies were found on detailed pediatric examination. Mental status was within normal limits for both siblings.

Heidelberg retinal tomography was performed and both eyes were within normal limits. Standard automated perimetry revealed minimal visual field loss and Seidel’s scotoma in both eyes.

**Figure 4:**
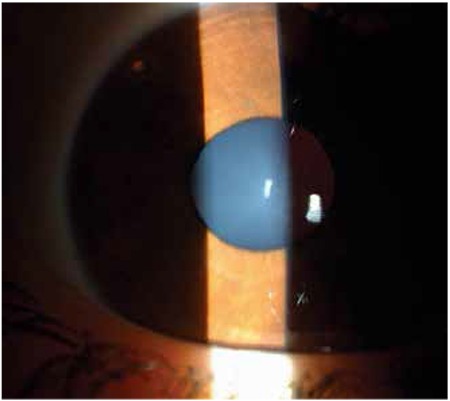
Anterior segment view of the patient after laser iridotomy

We initiated medical treatment of 0.5% timolol maleate drops applied twice a day to both eyes. IOP was 29 mmHg OD and 26 mmHg OS after one month of timolol maleate treatment. We then added 2% dorzolamide hydrochloride twice a day to the therapy. At final examination, IOP was 32 mmHg OD and 33 mmHg OS. Because of uncontrolled IOP, we performed laser iridotomy in both eyes (Figure 4). Gonioscopic and UBM examinations revealed open angle in both eyes after laser iridotomy.

After laser iridotomy, IOP was 21 mmHg OD and 23 mmHg OS. We prescribed 0.5% timolol maleate twice a day for both eyes. One month later, IOP was 15 mmHg OD and 16 mmHg OS. Her IOP levels were within normal limits at subsequent follow-up visits, but again increased to 38/24 mmHg OD/OS 15 months after laser iridotomy. The patient was given topical fixed-combination 0.5% timolol maleate 2% dorzolamide twice a day and 0.004% travoprost once a day. Following this treatment the IOP reduced to 32/19 mmHg OD/OS. Cyclopentolate 1% eye drops were administered three times a day to deepen the anterior chamber and correct appositional angle closure in order to reduce IOP. After this treatment her IOP values were 22/15 mmHg OD/OS. Although IOP was reduced to a safer level with cyclopentolate hydrochloride eye drops, the patient could not tolerate the treatment because of blurred vision. For this reason, clear lens extraction and intraocular lens implantation (IOL) was considered for the OD. The patient underwent phacoemulsification under general anesthesia, but an IOL could not be implanted because of small capsular bag and weak zonular support. Following lens extraction IOP reduced to 18 mmHg without antiglaucomatous medication. The patient began to use contact lens for refractive correction.

## DISCUSSION

Microspherophakia is usually associated with systemic disorders such as Weill-Marchesani syndrome (WMS), homocystinemia, Marfan syndrome, Alport syndrome and Klinefelter syndrome.^2,3,4,5,6,7,8,9,10^ Less commonly, it has been reported with other disorders such as Lowe syndrome, Peter’s anomaly, cri-du-chat syndrome, hyperlysinaemia, and rhizolemic form of chondrodysplasia punctata.^6,7,8,9^

Characteristic eye abnormalities of WMS are microspherophakia and ectopia lentis which causes high myopia (mostly dislocates either inferiorly or anteriorly). Other ocular associations are acute and/or chronic glaucoma, cataract and synechia. Glaucoma mostly develops due to the presence of the dislocated lens in the pupil or the anterior chamber. Progressive microspherophakia is responsible for severe and progressive myopia.^7,8,9^

Marfan syndrome is an autosomal dominantly inherited disorder. The main ocular features of Marfan syndrome, all of which can result in decreased vision, include bilateral lens dislocation, myopia and retinal detachment. About 80% of patients have ectopia lentis, which is usually bilateral, symmetrical and upward. The most prominent angle anomalies are dense iris processes and thickened trabecular sheets.^3,10^

Our patient had no clinical features to suggest any of these syndromes. Her mental status and height were normal. She had no cardiac, skeletal or muscular anomalies. Also, there was no evidence to suggest Alport syndrome in her pediatric examination. Her condition might be familial in origin, because her brother also has microspherophakia. Genetic counseling could not be performed due to the patient’s health insurance problem. Familial microspherophakia is not associated with any systemic defects. Although it is an autosomal recessive disorder, there is a case in the literature with autosomal dominant inheritance.^2^ Other ocular features of familial microspherophakia are lenticular myopia, posterior staphyloma, myopic crescent, ectopic pupil, glaucoma and retinal detachment.^2^

Glaucoma in isolated microspherophakia is not common.^2,3^ Several mechanisms can lead to glaucoma. Acute angle closure may result from pupillary block caused by the forward movement of the spherical lens or anterior chamber luxation of the lens due to weak and long zonules.^11^ Peripheral anterior synechiae formation by unrelieved pupillary block can cause synechial angle closure and irreversible trabecular meshwork damage. Chronic pupillary block may also lead to crowding of the anterior chamber angle by the spherical lens.^3,11^ Developmental anomaly of the anterior chamber angle may also contribute to the development of glaucoma in patients with microspherophakia.^3,11,12^

Our patient presented with bilateral angle-closure glaucoma. This resulted from pupillary block by the spherical lens. Peripheral iridectomy is the treatment of choice for these patients. Thus, uncontrolled IOP with medical therapy and resolution of her condition with laser iridotomy confirmed our diagnosis that microspherophakia induced pupillary-block glaucoma. However, these treatment modalities provided temporary improvement in the present case. For this reason, we suggest that more than one mechanism other than pupillary block might have led to the development of glaucoma in our case. As seen on UBM, the lens-iris diaphragm was persistently displaced anteriorally due to weak zonules and thus closed the iridocorneal angle. Therefore, IOP was not reduced permanently by laser iridotomy alone. Reduction of IOP after clear lens extraction confirms this observation. We did not consider lensectomy as a first choice therapy in our patient because of her age, risk of zonular defects, small capsular bag for standard IOL and postoperative visual problems such as accommodation and anisometropia. However, her IOP levels remained elevated despite patent peripheral iridotomy and maximum tolerated medical therapy, so we performed clear lens extraction. Indications for clear lens extraction in patients with microspherophakia are corneo-lenticular contact, unilateral high myopia, pupillary block and secondary intractable glaucoma.^12^ However, there are higher rates of intraoperative complications, such as difficulties performing capsulorhexis and implanting the IOL, during clear lens extraction of these patients. In our case, small capsular bag led to difficulty implanting the IOL into the bag. We did not implant the IOL into the sulcus because of the weakness of the zonules as this may be a risky situation for uncontrolled glaucoma.

Management of microspherophakia is still debated. Medical and laser treatment fail in about 60% of eyes with this condition. Lensectomy is still the first choice if medical therapy and laser iridotomy fail. There are reports in the literature of treating microspherophakia with lensectomy. Khokhar et al.^13^ reported a case that presented with superotemporally luxated microspherophakic lenses. They successfully treated this patient with clear lens extraction with IOL implantation. Kaushik et al.^12^ described an adult patient who presented with bilateral acute angle-closure glaucoma with microspherophakia and whose IOP was successfully controlled with lensectomy and anterior vitrectomy. Willoughby and Wishart^14^ described a case of spherophakia with glaucoma whose IOP was successfully controlled following lensectomy without additional medication. Kanamori et al.^15^ also described a patient with microspherophakia and chronic angle-closure glaucoma whose IOP was controlled well with goniosynechiolysis and lensectomy. In contrast, Yasar^16^ described a patient in whom IOP was not controlled with lensectomy in the short term and who subsequently required mitomycin-C augmented trabeculectomy in both eyes. Harasymowycz and Wilson^17^ advised a combination of lensectomy, anterior vitrectomy, scleral-fixated IOL and Molteno tube shunt implantation to control IOP in patients with uncontrolled chronic angle-closure glaucoma caused by microspherophakia. They concluded that in early cases, prophylactic laser iridotomy should be performed. Senthil et al.^18^ reported higher trabeculectomy success rates (86% at 6 months, 61% at 8 years) in patients with microspherophakia in their retrospective study with a long follow-up period. However, they observed significant complications, including shallow anterior chamber and iridocorneal and iridolenticular contact, which required surgical intervention. A large proportion (45%) of the trabeculectomized eyes underwent lensectomy for IOP control in their study.

In our study, lensectomy effectively decreased IOP, but continued follow-up is necessary to determine whether lensectomy alone will be sufficient for IOP control over a longer time period. Based on available data, we suggest that a stepwise treatment protocol would be more safe and effective in the management of the patients with glaucoma secondary to microspherophakia. According to this treatment protocol, laser iridotomy should be performed first. If laser iridotomy is ineffective, clear lens extraction with or without goniosynechiolysis, filtering surgery and tube shunt surgery may be performed, in that order.

In conclusion, optimal management of glaucoma in microspherophakia is still uncertain. Multiple factors are responsible for the development of glaucoma in microspherophakia. For this reason, success may not be obtained with a single treatment modality, as with our patient. Microspherophakic patients should be monitored closely to determine the appropriate method for treating their glaucoma.

### Ethics

Informed Consent: It was taken.

Peer-review: Externally peer-reviewed.
